# Dynamics of Cervical Lesions After Excisional Treatment in Relation to HPV Genotypes and Cytological Findings

**DOI:** 10.3390/jcm15031241

**Published:** 2026-02-04

**Authors:** Cornelius Eduard Carp, Alexandra Carp, Raluca Mihaela Gemanariu, Mihai Gabriel Marin, Sorana Caterina Anton, Handra Elicona, Alexandra Lazan, Raul Andrei Crețu, Emil Anton

**Affiliations:** 1 University of Medicine and Pharmacy “Grigore T. Popa”, 16 University Street, 700115 Iasi, Romania; andra1_c2003@yahoo.com (A.C.); raluca_gemanariu@yahoo.com (R.M.G.); marinmihai002@gmail.com (M.G.M.); elicona.handra@d.umfiasi.ro (H.E.); lazanalexandra@yahoo.com (A.L.); c.raulandy@yahoo.ro (R.A.C.); emil.anton@yahoo.com (E.A.); 2 Department of Gynecology, University of Medicine and Pharmacy “Grigore T. Popa”, 16 University Street, 700115 Iasi, Romania; 3 Department of Oral and Maxillofacial Surgery, University of Medicine and Pharmacy “Grigore T. Popa”, 16 University Street, 700115 Iasi, Romania; 4 “Elena Doamna” Clinical Hospital of Obstetrics and Gynecology, 49 Elena Doamna Street, 700398 Iasi, Romania; 5 Department of Mother and Child Medicine, University of Medicine and Pharmacy “Grigore T. Popa”, 16 University Street, 700115 Iasi, Romania; 6 Clinical Hospital of Obstetrics and Gynecology “Cuza Voda”, 700038 Iasi, Romania; 7 Neurology Department, University of Medicine and Pharmacy “Grigore T. Popa”, 16 University Street, 700115 Iasi, Romania

**Keywords:** cervical intraepithelial neoplasia, Papanicolaou test, colposcopy, conization, cervical cytology, histopathology, cervical biopsy, low-grade squamous intraepithelial lesion (LSIL), high-grade squamous intraepithelial lesion (HSIL)

## Abstract

**Background/Objectives:** Human papillomavirus (HPV) infection remains the principal etiologic factor for cervical intraepithelial neoplasia (CIN) and cervical cancer. This longitudinal cohort study aimed to characterize the dynamics of cytological and histopathological changes over a two-year follow-up, focusing on post-treatment reduction in lesion grade, persistence, and progression in relation to HPV genotype distribution and smoking status. **Methods:** A total of 351 women aged 20–76 years were included, with cervical samples collected at the “Elena Doamna” Clinical Hospital, Iași, Romania. Cytology was categorized according to the Bethesda System, while colposcopy and conization served as diagnostic confirmation methods. HPV genotyping identified both high-risk (HR) and low-risk (LR) viral subtypes. Longitudinal assessments were performed at baseline, one-year, and two-year intervals to evaluate temporal patterns of disease evolution. **Results:** At baseline, HSIL represented the predominant cytologic category (51.3%, n = 180), followed by ASC-US (19.1%), ASC-H (15.1%), and LSIL (14.5%). Negative cytology increased from 62.4% at one year to 71.8% at two years, indicating substantial post-treatment reduction in lesion grade. Downgrading of lesion severity after treatment occurred in 26.2%, persistence in 11.1%, and progression in 11.1% of cases. Concordance between colposcopy and conization was moderate but statistically significant (κ = 0.345), with the highest agreement observed for HSIL with equivocal features between CIN II and CIN III lesions. Smoking showed a significant association with lesion persistence at two years (OR = 3.07; 95% CI: 1.16–8.08) but no statistically significant association with HR-HPV persistence. HR-HPV genotypes 16, 18, 31, and 33 were most frequently linked to progression, whereas HPV 35, 59, and 68 were associated with persistence. **Conclusions:** Over two years, most cervical lesions regressed or normalized, demonstrating effective management and follow-up. Persistent infection with HR-HPV types and smoking were the primary determinants of unfavorable outcomes. These findings highlight the clinical relevance of sustained surveillance, HPV genotyping, and smoking cessation as integral components of evidence-based cervical disease prevention and management strategies.

## 1. Introduction

Persistent high-risk human papillomavirus (HR-HPV) infection accounts for more than 95% of cervical cancer cases [1,2,3]. Although most human papillomaviruses (HPV) infections clear spontaneously within 1–2 years, approximately 10–20% persist, substantially increasing the risk of cervical and other anogenital cancers [4,5,6,7,8]. The global age-standardized incidence rate of cervical cancer is 13.1 per 100,000 women, with a mortality rate of 6.9 per 100,000 [6,7,8,9]. Cervical cancer continues to cause significant morbidity and mortality, particularly in low- and middle-income regions with limited access to preventive services [10]. While cytology-based screening has historically reduced incidence and mortality, its limited sensitivity has led to the implementation of primary HPV DNA testing, which offers superior detection of high-grade precancerous lesions [10,11]. HPV-related disease is also associated with important psychosocial consequences, including stigma, anxiety, and fear of disclosure [12,13,14].

Despite widespread screening, important gaps remain in the understanding of HPV-related disease dynamics, age-specific genotype distribution, and the concordance between cytological, colposcopic, and histopathological findings. Epidemiological data indicate that 80–95% of sexually active individuals will acquire HPV during their lifetime [15,16]. While most infections resolve spontaneously, approximately 10% persist and may progress to high-grade lesions or invasive cancer. Viral oncogenes promote persistence through immune evasion, while cofactors such as smoking, coinfections, long-term oral contraceptive use, multiparity, nutritional deficiencies, and immunosuppression further increase the risk of progression from low-grade squamous intraepithelial lesion (LSIL) to high-grade squamous intraepithelial lesion (HSIL) and invasive carcinoma [6,9,17,18].

HPV remains the most common sexually transmitted viral infection worldwide, with prevalence estimates ranging between 75% and 95% among sexually active individuals [12,18]. Considerable geographic variation exists in HPV prevalence, genotype distribution, and cancer burden [1,19,20,21,22,23,24,25]. Globally, over 630 million people are affected by HPV, and the virus is responsible for approximately 4.5–5.2% of all cancers worldwide, corresponding to nearly 630,000 new cases annually [26,27,28,29,30]. While nearly all cervical cancers are HPV-related [5,31], the virus also contributes substantially to anal, vaginal, vulvar, penile, and oropharyngeal cancers, with emerging associations reported for sinonasal and ocular tumors [31,32]. The highest burden of HPV-related cancers occurs in low- and middle-income countries, where nearly 90% of cervical cancer deaths are reported [14,33,34,35,36].

Given the central role of HPV in cervical carcinogenesis, characterization of genotype-specific distribution, persistence, and lesion evolution is essential for optimizing screening strategies and clinical management [1,5,6,7,8,9,10,11]. The present study was designed to characterize baseline HPV genotype distribution in a cohort of 351 patients, evaluate age-related prevalence of HR-HPV and low-risk human papillomavirus (LR-HPV), and monitor lesion progression and post-treatment reduction in lesion grade at 6 months, 1 year, and 2 years of follow-up. Additional objectives included assessing concordance between Papanicolaou test results, colposcopic findings, and conization outcomes, as well as evaluating long-term lesion behavior following diagnostic and therapeutic interventions [37,38,39,40,41,42,43,44,45,46,47,48,49].

Because smoking represents a well-established behavioral cofactor in cervical carcinogenesis, its association with HPV persistence and lesion progression was also examined. Smoking status was recorded dichotomously, reflecting its clinical relevance and biological plausibility as a contributor to HPV-related disease progression. This integrated approach aimed to define genotype-specific trajectories, diagnostic concordance, and risk factor associations in the post-treatment course of cervical lesions.

## 2. Materials and Methods

We conducted a prospective longitudinal cohort study to evaluate the post-excisional dynamics of HPV infection and cervical lesions over 24 months, focusing on the relationship between high-risk and low-risk HPV genotypes and cytological and histological outcomes across age groups. A longitudinal design was chosen to capture genotype-specific persistence, clearance, progression, and downgrading of lesion severity after treatment over time, model time-to-event outcomes, and minimize recall bias relative to cross-sectional designs. The study was conducted at the “Elena Doamna” Clinical Hospital of Obstetrics and Gynecology, Iași, Romania, where patient recruitment and sample collection were carried out. HPV testing was performed in private accredited laboratories, and all cytological data were recorded and analyzed for follow-up evaluation. Enrollment took place between January 2020 and September 2023, with each participant followed for a minimum of 24 months. The study concluded in August 2025 after completion of follow-up for the last enrolled patients.

Female patients presenting for routine cervical cancer screening or diagnostic evaluation at the “Elena Doamna” Clinical Hospital of Obstetrics and Gynecology, Iași, were eligible for enrollment. Participants had to be adults (over 18 years), able to provide written informed consent, undergo baseline Pap cytology and HPV DNA testing, and agree to scheduled follow-up visits. In line with the study objectives, only women with confirmed HPV positivity at baseline were included.

Women were excluded if they lacked written informed consent, had undergone a prior total hysterectomy, provided inadequate or insufficient cervical samples at baseline, or presented cytological specimens deemed unsatisfactory or inconclusive for evaluation. Women with normal cytology (NILM) at baseline were also excluded, as only patients with abnormal cytological findings were eligible for inclusion in order to justify colposcopic evaluation, biopsy, and longitudinal lesion follow-up. Additional exclusion criteria included a known history of invasive cervical cancer prior to enrollment, missing essential identifiers that prevented longitudinal follow-up, pregnancy at the time of enrollment, immunocompromised status (such as HIV infection or chronic immunosuppressive therapy), and any prior cervical treatments or procedures that could modify baseline lesion characteristics or influence post-excisional outcomes. Women who were HPV-negative at baseline or who did not adhere to the scheduled follow-up protocol were also excluded from the final analysis.

To ensure consistent and clinically meaningful classification of lesion evolution after treatment, outcomes were defined using combined cytological, colposcopic, and histological criteria, as follows:

Clearance (absence of residual disease/post-treatment cytological normalization): was defined as the absence of detectable disease after treatment, corresponding to NILM and/or no colposcopic or histological evidence of cervical intraepithelial neoplasia (CIN) at follow-up.

Persistence: was defined as the maintenance of the same cytological or histological lesion grade at follow-up compared with the previous evaluation.

Progression: was defined as an increase in lesion severity over time, expressed as worsening cytological abnormalities or a higher CIN grade on colposcopy or histology.

Post-treatment downgrading of lesion severity: was defined as a decrease in lesion severity after treatment, reflected by a lower cytological category or a reduced CIN grade on follow-up.

### 2.1. Recruitment and Consent

Potentially eligible patients were identified consecutively from screening clinics and colposcopy referrals. A trained study nurse obtained written informed consent and administered a brief baseline questionnaire. Participants received unique study IDs, and personal identifiers were stored separately from research data. Participants were scheduled for visits at baseline (month 0), 6 months, 12 months, and 24 months.

### 2.2. Data Collection and Clinical Workflow

The study protocol included a longitudinal follow-up of all HPV genotypes detected at baseline. Each identified HPV type was tracked across subsequent evaluations in order to assess viral persistence, clearance, and its relationship with cytological and histopathological evolution following excisional treatment. This design allowed genotype-specific risk profiling and facilitated correlation between viral behavior and clinical outcomes.

Cervical lesions identified during the study were classified according to the Bethesda System for cervical cytology, which provides internationally accepted diagnostic categories for Pap smears. The following categories were used in our study:

- Negative for intraepithelial lesion or malignancy (NILM)
- Atypical squamous cells of undetermined significance (ASC-US)
- Low-grade squamous intraepithelial lesion (LSIL)
- Atypical squamous cells, cannot exclude HSIL (ASC-H)
- High-grade squamous intraepithelial lesion (HSIL)
- Atypical glandular cells (AGC) [42,43]

Cytological results were recorded at each scheduled visit and were used to determine the need for further diagnostic procedures (Table 1).

### 2.3. Colposcopic Assessment

All patients with abnormal cytology results (≥ASC-US), as well as those with persistent HR-HPV infection regardless of cytology grade, were referred for colposcopic evaluation. Colposcopy was performed using standardized protocols, with directed biopsies taken from areas with acetowhite changes, punctation, mosaic patterns, or atypical vascularization. Colposcopy results were integrated with cytology findings to guide management decisions and to provide histological confirmation of cervical intraepithelial neoplasia (CIN).

### 2.4. Histological Confirmation and Conization

Biopsies obtained during colposcopy were processed by the pathology department and classified as CIN I, CIN II, CIN III, or invasive carcinoma, according to the World Health Organization (WHO) criteria [45]. In cases where the distinction between CIN II and CIN III was uncertain and the pathology report stated “cannot exclude CIN III,” lesions were classified as HSIL with equivocal features between CIN II and CIN III for the purpose of longitudinal evaluation. Although CIN I is typically managed conservatively according to international guidelines, in the present study conization was also performed in selected CIN I cases (n = 3). This decision was based on clinical indications such as discordant cytology-colposcopy findings, HSIL or ASC-H cytology with biopsy showing CIN I, uncertainty regarding lesion grade (“cannot exclude CIN II/III”), non-visualization of the squamocolumnar junction, and the need for complete excisional assessment to ensure an accurate diagnosis. Additionally, excisional procedures were required to allow precise longitudinal evaluation of lesion evolution within the study protocol. Therefore, all patients included in the cohort underwent conization for diagnostic and/or therapeutic purposes [44,45].

### 2.5. Management During Follow-Up

Clinical management (repeat cytology, HPV testing, colposcopy, excisional treatment) followed Romanian and international guidelines applicable during 2020–2025.

In our cohort of 351 patients, the only cytological categories identified were ASC-US, LSIL, ASC-H, and HSIL; therefore, only these diagnostic groups were included in the present analysis. Cervical specimens were collected at baseline (month 0), 6 months, 12 months, and 24 months. HPV genotyping was performed at baseline, 12 months, and 24 months. DNA extraction and HPV genotyping were conducted using two commercially available assays: the Anyplex^TM^ II HPV28 Detection kit (Seegene Inc., Seoul, Republic of Korea), a real-time multiplex PCR-based assay, and the High & Low PapillomaStrip assay (Operon, Zaragoza, Spain), a PCR-based reverse hybridization method, according to the manufacturers’ instructions. The Anyplex^TM^ II HPV28 assay enables the detection of the following high- and intermediate-risk HPV genotypes: 16/18/26/31/33/35/39/45/51/52/53/56/58/59/66/68/69/73, and 82, as well as the low-risk genotypes HPV 6/11/40/42/43/44/54/61, and 70. The High & Low Papilloma Strip assay detects high-risk genotypes HPV 16/18/26/31/33/35/39/45/51/52/53/56/58/59/66/68/69/73, and 82, and low-risk genotypes HPV6/11/40/42/43/44/54/61/62/67/70/71/72/74/81/83/84, and 91. The combined use of two complementary, clinically validated HPV genotyping assays increased the robustness of genotype detection and minimized the risk of false-negative results. Persistence, clearance, and acquisition of individual HPV genotypes were tracked longitudinally across study visits.

Colposcopy was performed at baseline and follow-up, with excisional procedures (including conization) when clinically indicated. All colposcopic and histopathological findings were systematically recorded. Lesion evolution was assessed in relation to HPV genotype and major risk factors, including smoking, recorded dichotomously. Cytology, HPV genotyping, colposcopy, and histopathology were linked using a unique study identifier, enabling both baseline and longitudinal analyses of genotype-specific lesion outcome

### 2.6. Statistical Analysis

All analyses were performed using IBM SPSS Statistics (version 26.0). Descriptive statistics were used to summarize demographic, clinical, and virological characteristics, with categorical variables reported as frequencies and percentages. Distributional assumptions and linearity were evaluated prior to analysis to guide the selection of appropriate parametric or non-parametric tests. Associations between categorical variables, including smoking status, lesion persistence, progression, cytological outcomes, and histologic findings, were primarily assessed using Pearson’s chi-square test or Fisher’s exact test, as appropriate. Cross-tabulation analyses were used to explore the distribution and concordance of lesion grades between cytology, colposcopy, and conization, particularly for CIN categories. Longitudinal changes in cytological severity across baseline, 1-year, and 2-year follow-up were analyzed using the Friedman test, with pairwise comparisons performed by Wilcoxon signed-rank tests. Effect size for repeated measures was estimated using Kendall’s W. Binary logistic regression was applied to evaluate the association between smoking and lesion persistence or progression at 1 and 2 years, with results expressed as odds ratios (ORs) and 95% confidence intervals (CIs). Model performance was assessed using Omnibus tests of model coefficients. For outcomes with a limited number of events, only univariate estimates were reported. Linear-by-linear association tests were used to assess ordered trends, and agreement between categorical diagnostic classifications was evaluated using Cohen’s kappa coefficient. All statistical tests were two-sided, and a *p*-value < 0.05 was considered statistically significant.

## 3. Results

**Figure 1:**
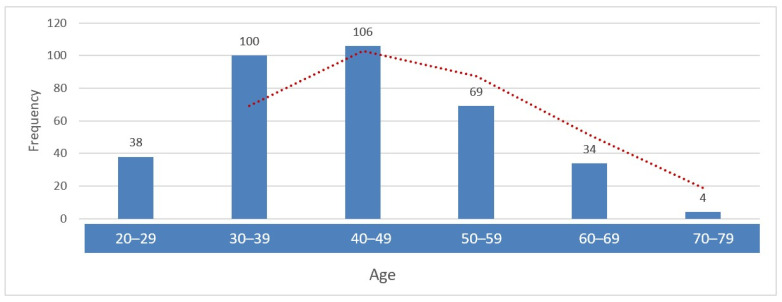
Age group distribution of the study cohort (20–76 years).

A total of 351 female patients aged 20–76 years were included in the study. The mean age was 43.8 ± 11.4 years, with a median of 43 years and a mode of 44 years. The interquartile range (35–51 years) indicated that most participants were concentrated in the midlife decades. For analysis, age was categorized into 10-year intervals. The largest proportion of women were aged 30–49 years (58.7%, n = 206). Women aged 20–29 years accounted for 10.8% (n = 38), those aged 50–59 years for 19.7% (n = 69), women aged 60–69 years for 9.7% (n = 34), and only four participants (1.1%) were aged 70 years or older (Figure 1).

**Figure 2:**
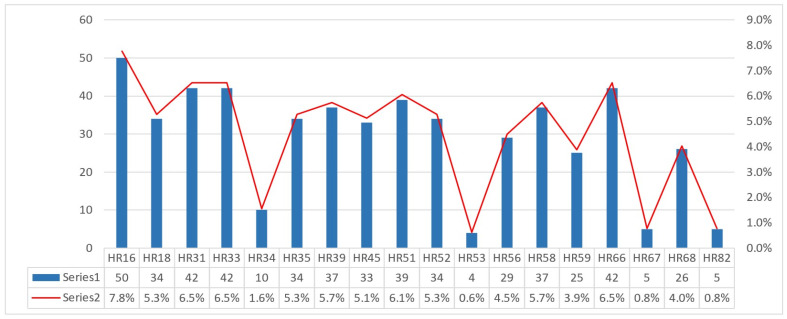
Frequency distribution of HR-HPV genotypes in the study cohort.

At baseline, smoking prevalence was high, with 249 women (70.9%) being current smokers, and was therefore retained as a key covariate in all subsequent analyses. Cytological assessment at enrollment revealed a predominance of high-grade abnormalities, with HSIL identified in 180 women (51.3%), indicating an advanced disease profile at study entry (Table 1). HPV testing demonstrated frequent multiple-genotype infections, with both HR and LR types represented. Among HR-HPV, several oncogenic genotypes predominated, most notably HPV16/31/33/66, and 51, while additional HR types occurred at intermediate frequencies (Figure 2).

**Figure 3:**
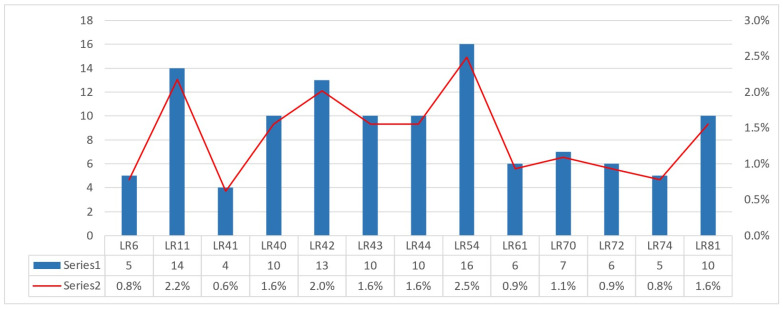
Frequency distribution of LR-HPV genotypes in the study cohort.

LR-HPV genotypes were less prevalent and more heterogeneously distributed, with only a few types accounting for most LR detections and the remainder occurring sporadically (Figure 3).

### 3.1. Evolution of Lesions

A Friedman test demonstrated significant differences in Pap smear cytological lesion severity across baseline, 1-year, and 2-year evaluations (*p* < 0.001), indicating a consistent downward trend. Mean ranks decreased from 2.90 at baseline to 1.65 at 1 year and 1.45 at 2 years, confirming overall post-treatment reduction in lesion grade. Paired Wilcoxon tests showed significant improvement of cervical lesions from baseline to 1 year (Z = −15.27, *p* < 0.001) and from baseline to 2 years (Z = −16.05, *p* < 0.001).

At the 1-year follow-up, 8 women (2.3%) showed true progression, 35 (10.0%) demonstrated persistence, and 308 (87.7%) showed downgrading of lesion severity after treatment, as assessed by cytological criteria. Progression involved multiple HPV genotypes, predominantly high-risk types. The most frequent were HPV16 and HPV39 (each 0.9%), followed by HPV51 (0.6%), while other isolated high-risk types included HPV31/33/52/56/59. Among low-risk types, progression occurred with HPV6/42/70 (each 0.3%). Persistence at 1 year showed a broader high-risk distribution, dominated by HPV66 (2.6%), HPV39 (2.0%), HPV51 (1.7%), and HPV16 (1.4%), with additional involvement of HPV56/68/58/18/35/45/59. Low-risk persistence involved HPV11/41/42/74. Chi-square, correlation, and regression analyses showed no overall significant association between genotype and lesion evolution; however, HPV44 and HPV66 were individually associated with progression, and HPV66 with persistence.

At the 2-year evaluation, post-treatment reduction in lesion grade was observed in 92 cases (26.2%), while persistence and progression were each identified in 39 cases (11.1%). Persistent abnormalities were significantly associated with HPV35/59/68, whereas progression was linked to HPV16/18/31/33, representing the most frequent high-risk genotypes associated with unfavorable outcomes at two years.

### 3.2. Clearance

Among the 351 women included in the study, HSIL were the most frequent cytological finding, identified in 180 cases (51.3%) (Table 2).

The highest proportion of cytological abnormalities occurred in women aged 40–49 years (30.2%), followed by those aged 30–39 years (28.5%) and 50–59 years (19.7%), together accounting for nearly 80% of all abnormal findings. In the 40–49-year group, HSIL was the predominant category (57.5%), while in women aged 30–39 years HSIL was identified in 50%. Among women aged 20–29 years, HSIL also predominated (44.7%), followed by LSIL (26.3%) and ASC-US (13.2%). In older age groups, although the overall frequency of abnormalities declined, HSIL remained proportionally frequent, accounting for 47.8% in women aged 50–59 years, 50% in those aged 60–69 years, and 50% in women aged ≥70 years (Table 2).

At the 1-year evaluation, 62.4% of women had negative cytology, while 37.6% still showed abnormalities. ASC-US was the most frequent abnormal category (22.8%), followed by ASC-H (9.7%), LSIL (3.1%), and HSIL (2%) (Table 2).

At the 2-year follow-up, cytological abnormalities persisted in 28.2% of women, predominantly ASC-US (25.1%) and ASC-H (3.1%). No LSIL or HSIL cases were observed at this time point. Abnormal findings were more frequent in women aged 30–39 and 40–49 years, whereas in the remaining age groups most women had negative cytology (Table 2).

A comparative analysis between colposcopic impressions and conization histology showed a moderate but statistically significant agreement (Cohen’s κ = 0.345, *p* < 0.001), with full concordance in 192 of 351 cases (55%). Agreement was highest for CIN II, CIN III, and HSIL with equivocal features between CIN II and CIN III (all *p* < 0.001). (Table 3).

For CIN II, exact concordance was observed in 64 cases (*p* < 0.001), while 16 lesions initially classified as CIN II by colposcopy were downgraded to CIN I after conization (*p* < 0.001). For CIN III, conization confirmed 85 cases (*p* < 0.001); however, 16 cases were reclassified as CIN II and 12 as HSIL. No lesions initially classified as CIN I by colposcopy were confirmed at conization; all were instead upgraded to CIN III (*p* < 0.001). The HSIL category showed 43 concordant cases (*p* < 0.001). Overall, colposcopy more frequently overestimated lesion severity (approximately 17%) than underestimated it (approximately 9%). The Chi-square test (df = 9; *p* < 0.001) confirmed a non-random distribution, supporting consistent diagnostic performance particularly for CIN II, CIN III, and equivocal HSILs (Table 3).

A comparative assessment between cytology, colposcopy, and conization revealed consistent diagnostic patterns across lesion severities (Table 4). Low-grade cytology (ASC-US and LSIL) was frequently associated with high-grade disease at subsequent evaluation, indicating a tendency of cytology to underestimate lesion severity.

Among ASC-US cases, colposcopy identified CIN II-III in 95.5% of women, while conization confirmed CIN II+ lesions in 77.6%, supporting the clinical relevance of this category. Similarly, in ASC-H cases, 84.9% were confirmed as CIN II or higher at histology (*p* < 0.001). LSIL cytology also showed a high rate of upgrading, with 94.1% of cases classified as CIN II+ on conization, emphasizing the limited reliability of LSIL for excluding significant disease. HSIL cytology demonstrated the strongest concordance with high-grade lesions: 99% of HSIL cases corresponded to moderate or severe lesions at colposcopy, and 98.3% were confirmed as CIN II+ at conization (*p* < 0.001), highlighting its high predictive value.

Overall, concordance between cytology, colposcopy, and excisional histology increased with lesion severity, confirming a progressive alignment across diagnostic modalities (Table 4).

Smoking was not associated with lesion persistence at 1 year, when smokers represented 74.3% of persistent cases compared with 70.6% of non-persistent cases (*p* = 0.646). At 2 years, smoking became significantly associated with persistence, with smokers accounting for 87.2% of persistent cases versus 68.9% of non-persistent cases (*p* = 0.018; Fisher’s *p* = 0.023) (Table 5).

Smoking was also significantly associated with lesion progression. At 1 year, smokers represented 25.0% of progression cases compared with 72.0% in the non-progression group (*p* = 0.004), and at 2 years smokers accounted for 53.8% of progression cases versus 73.1% of non-progression cases (*p* < 0.05).

At baseline, smokers exhibited a higher burden of cytological abnormalities, with ASC-US observed in 26.1% of smokers versus 14.7% of non-smokers and ASC-H in 11.6% versus 4.9%, respectively (*p* = 0.013), indicating greater cytological severity among smokers. At the 2-year follow-up, cytological outcomes no longer differed significantly by smoking status. Regression analyses confirmed that smoking was associated with lesion persistence at 2 years (OR = 3.07, *p* = 0.023) and with progression risk (OR = 0.43, *p* = 0.015), although the explained variance was modest. HR-HPV persistence remained numerically higher among smokers at both follow-ups (48.6% vs. 42.2% at 1 year; 22.9% vs. 15.7% at 2 years), without reaching statistical significance (Table 5).

## 4. Discussion

In 2020, cervical cancer accounted for 604,127 new cases and 341,831 deaths worldwide, underscoring its persistent global impact [6,46,47,48]. In our cohort, post-treatment reduction in lesion grade was the predominant outcome, with 71.8% of women showing normal cytology at two years, while mild abnormalities (mainly ASC-US) persisted in 25.1% and only 3.1% showed ASC-H, with no remaining HSIL. This distribution reflects the dynamic course of HPV-related disease, where most lesions regress and a minority persist, as also reported by recent studies highlighting the role of biomarkers and viral-load kinetics in lesion evolution [49,50,51]. Approximately one in five women experienced persistence or progression, consistent with reported HR-HPV persistence rates of 20–25% beyond 12–24 months [52]. These findings emphasize persistence, rather than initial detection, as the key driver of clinical risk and follow-up intensity in HPV-positive women.

### 4.1. Principal Findings of the Study

**Figure 4:**
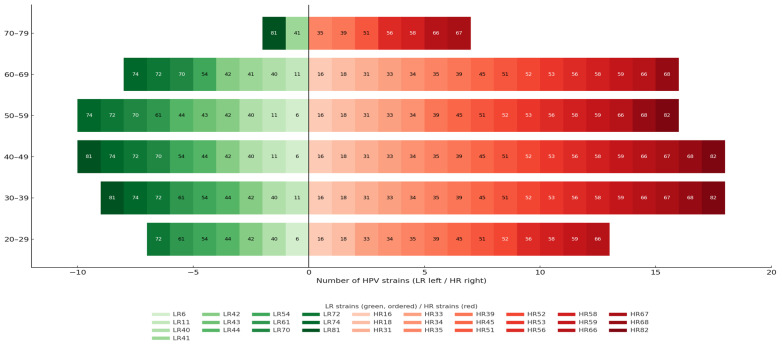
Distribution of LR- and HR-HPV strains by age group.

HSIL prevalence peaked between ages 30 and 49, while women aged 20–29 showed a predominance of LSIL (Figure 4).

Although fewer in number, women over 50 exhibited more severe and persistent lesions. This distribution is biologically plausible, as younger women clear HPV more efficiently, whereas advancing age is associated with immunosenescence and cumulative exposure to cofactors that favor persistence and progression. The predominance of HR-HPV in this cohort underscores the substantial oncogenic potential of the study population and supports the need for surveillance focused on HR-HPV-positive women, as well as genotype-specific analyses.

In this cohort, HR-HPV prevalence peaked in women aged 30–49 years, while LR-HPV types remained infrequent across all age groups. HPV16/31/33/66, and HPV51 were the most frequently detected HR genotypes at baseline, reflecting a high burden of oncogenic HPV infections in the study population.

Lesion evolution showed a predominant trend toward downgrading of lesion severity after treatment and healing, with 87.7% of cases regressing at 1 year and more than 70% demonstrating post-treatment reduction in lesion grade or complete healing at 2 years. Nevertheless, persistence and progression were observed in a clinically relevant proportion of women (approximately 11% each at 2 years), underscoring the heterogeneous course of HPV-related cervical disease.

Distinct HPV genotypes were associated with unfavorable outcomes. At 1 year, persistence was most frequently linked to HPV39/66/51/16, while progression involved mainly HPV16/39. At 2 years, persistent abnormalities were significantly associated with HPV35/59/68, whereas true progression correlated with HPV16/18/31/33, identifying these genotypes as those most strongly linked to long-term adverse outcomes in our cohort.

The best diagnostic agreement between colposcopy and excisional histology was observed for CIN II, CIN III, and HSILs with equivocal CIN II-III features, which showed the highest rates of concordance between the two methods. In contrast, lesions initially classified as CIN I by colposcopy showed poor concordance, as most of these were upgraded to CIN II, CIN III, or HSIL at conization. Overall, colposcopy more frequently overestimated lesion severity than underestimated it, highlighting that discrepancies between the two techniques were mainly driven by low-grade colposcopic assessments rather than high-grade lesions.

Smoking emerged as a relevant modifier of lesion evolution. Although smoking was not associated with persistence at 1 year, it was significantly associated with persistence and progression at 2 years. Smokers also exhibited a higher proportion of abnormal cytological findings and a numerically higher rate of HR-HPV persistence throughout follow-up, suggesting a detrimental effect of smoking on lesion clearance.

### 4.2. Comparison with Existing Literature and Contextualization of Findings

The predominance of post-treatment reduction in lesion grade observed in our cohort is concordant with current biological models of HPV-related lesion evolution. Li et al. (2024) [49] showed that molecular and epigenetic biomarkers can distinguish CIN2 lesions prone to downgrading of lesion severity after treatment from those more likely to progress, supporting the existence of measurable biological differences between transient and persistent infections [46,47,48,49]. Similarly, Zamurovic et al. (2023) [50] emphasized the importance of viral-load kinetics in understanding lesion behavior, while Tessandier et al. (2025) [51] demonstrated that non-persistent infections typically undergo a plateau phase of 13–20 months before rapid clearance, providing a biological explanation for prolonged low-grade persistence prior to healing [50,51,52]. These findings are consistent with our results, in which post-treatment reduction in lesion grade was the most frequent outcome, whereas persistence defined a smaller but clinically relevant high-risk subgroup.

Our genotype-specific observations further align with prior evidence. Wang et al. (2025) [53] demonstrated that among women with ASC-H or HSIL cytology, those infected with HPV16 or HPV18 had a significantly higher risk of CIN3+ compared with women harboring other high-risk types. This is in agreement with our findings, where HPV16, HPV18, HPV31, and HPV33 were most strongly associated with progression at two years, underscoring the central role of specific oncogenic genotypes in determining disease severity.

Age-related patterns observed in our cohort are also supported by population-level data. A 2024 BMC Women’s Health study confirmed that CIN2+ lesions cluster predominantly among women aged 30–49 years, with the greatest burden in HPV16/18-positive cases [54,55]. Although women over 50 represented a smaller proportion of our cohort, they exhibited more persistent and severe lesions, reinforcing WHO recommendations to maintain cervical screening beyond age 50, particularly in previously underscreened women [56]. Clinically, this highlights the need for continued vigilance in postmenopausal women, a group increasingly affected by underdiagnosed cervical cancer [57].

With regard to diagnostic performance, Li et al. (2023) [54] reported a colposcopic accuracy of 87.4% for CIN2+, with sensitivity of 50.5% and specificity of 93.7%, and noted frequent underestimation of lesion grade, particularly in high-risk patients. A meta-analysis yielded similar estimates, with pooled accuracy around 89% and specificity near 93% [58]. Retrospective conization studies likewise demonstrated that concordance improves with increasing lesion severity but remains variable for mild abnormalities [59,60]. These observations closely mirror our results, where diagnostic alignment was strongest for CIN II, CIN III, and equivocal HSILs, while low-grade lesions posed the greatest diagnostic challenges.

Several studies support the observation that smokers have approximately a twofold higher risk of persistent HPV infection and CIN progression compared with non-smokers [61,62,63]. Smoking-related findings in our study are also consistent with current mechanistic and epidemiologic evidence. The Catalán-Castorena review (2024) [63] highlighted that HR-HPV integration is facilitated by cellular microenvironment perturbations, including those induced by smoking. Moreover, molecular biomarker studies suggest that smokers with hrHPV are more likely to exhibit methylation patterns predictive of progression. Although smoking was not significantly associated with persistence at one year in our cohort, it became a predictor of both persistence and progression at two years, likely reflecting the cumulative effects of oxidative stress, DNA damage, and immune suppression induced by chronic tobacco exposure [63,64,65,66]. Consistent with these reports, our findings further indicate a time-dependent effect of smoking on lesion evolution. In our cohort, although smoking status was assessed categorically, a significant association emerged over longer follow-up intervals: smoking was not linked to persistence at one year but became significantly associated with both persistence and progression at two years. This delayed association supports the hypothesis that the carcinogenic and immunomodulatory effects of tobacco require prolonged exposure to exert a measurable impact on HPV-related lesion behavior, in line with evidence showing cumulative oxidative stress, DNA damage, and impaired viral clearance among smokers.

An important frontier in cervical disease management is the development of multimodal predictive models combining cytology, HPV genotype, molecular biomarkers (p16/Ki67, methylation assays), inflammation markers, and clinical risk factors. He et al. (2024) [67] developed AI-driven models for predicting LSIL downgrading of lesion severity after treatment and progression by integrating patient demographics, HPV status, and pathological image features. On the viral dynamics front, Sierra-Rojas et al. (2022) [68] proposed mathematical models describing HPV persistence, clearance, and lesion transitions as probabilistic processes shaped by host and viral factors. Recent advances in predictive modeling highlight the value of integrating cytology, HPV genotyping, and molecular biomarkers to improve risk stratification in cervical disease [66,67,68]. In line with these developments, AI-assisted cytology and colposcopy have demonstrated high diagnostic accuracy and the potential to reduce interobserver variability, consistent with the moderate concordance observed in our study. Traditional cytology-HPV paradigms are thus increasingly complemented by biomarker-based and AI-assisted approaches to refine risk stratification. A 2024 eClinicalMedicine meta-analysis showed that AI-assisted cytology and colposcopy achieved accuracy of 94%, sensitivity of 95%, and specificity of 94%, comparable or superior to expert clinicians [69]. Integration of such technologies may enhance diagnostic consistency and address the moderate interobserver agreement observed in our study, reinforcing the concept that lesion evolution is not deterministic but probabilistic, governed by complex interactions between viral genotype, host factors, immune response, and microenvironment.

### 4.3. Clinical Implications

The strong correspondence observed between colposcopic impressions, excisional histology and lesion severity, particularly for CIN II, CIN III and equivocal HSILs, confirms that colposcopy-guided decision-making remains reliable for identifying women requiring excisional treatment. In contrast, lower-grade abnormalities showed greater diagnostic variability, reinforcing the need for cautious interpretation and structured follow-up in these categories rather than immediate intervention.

The longitudinal follow-up demonstrated that lesion evolution is not uniform and should be interpreted in relation to both time and HPV genotype. The clear association between persistent high-risk HPV, especially HPV16 and HPV18, and unfavorable outcomes supports genotype-based risk stratification as a practical tool in routine care. In this context, HPV genotyping adds clinically actionable information beyond cytology alone, allowing earlier identification of women who may benefit from intensified surveillance or timely therapeutic intervention.

Equally important, the high rate of post-treatment reduction in lesion grade observed within the first two years, particularly among women without persistent high-risk HPV, supports conservative management strategies in selected patients, avoiding unnecessary excisional procedures and their potential reproductive and obstetric consequences. Conversely, lesions persisting beyond 24 months represent a clinically relevant subgroup with higher progression risk, indicating that persistence rather than initial lesion grade should guide escalation of management.

The delayed but significant association between smoking and long-term persistence and progression highlights smoking status as a modifiable cofactor that should be systematically incorporated into risk assessment and follow-up planning. From a clinical standpoint, this supports the inclusion of smoking cessation counseling as an integral component of HPV-related disease management.

HPV vaccination, including bivalent, quadrivalent, and 9-valent formulations, is highly effective, with the nonavalent vaccine offering the broadest protection [6,25]. While most HPV infections clear spontaneously, persistent infection drives the majority of HPV-related morbidity, with HPV16 and HPV18 responsible for most cervical cancers [14,29,70,71,72,73]. HPV DNA testing remains the cornerstone of screening, supported by cytology as needed [74,75,76,77,78,79], although global vaccination coverage remains insufficient to fully curb oncogenic HPV circulation [80,81,82,83,84,85].

### 4.4. Limitations

A methodological limitation of this study relates to the diagnostic category “HSIL with equivocal features between CIN II and CIN III.” Although not an officially standardized histopathological classification, this terminology reflects a common real-world scenario in which pathologists report lesions as “CIN II, cannot exclude CIN III.” Rather than forcing these ambiguous cases into rigid categories, we retained an equivocal classification to accurately represent diagnostic uncertainty and to allow clearer longitudinal tracking of lesion evolution after excisional treatment.

A second limitation concerns the handling of excisional margin status. Although margin assessment was routinely performed by the pathology laboratory, the excised specimens were not consistently received as intact, orientable surgical pieces. Because conizations originated from multiple operators and involved variable techniques, specimen fragmentation and inconsistent orientation prevented uniform and reliable margin interpretation. As a result, margin status could not be collected in a standardized form for integration into the study database. Given that incomplete or non-comparable margin data would have introduced bias rather than strengthening the analysis, margin status was intentionally not included as an analytical variable. Although most reports suggested negative margins, the lack of uniform reporting standards and frequent specimen fragmentation precluded meaningful stratification or statistical analysis based on margin status. The last limitation of the present study concerns the assessment of smoking exposure. Although smoking status (smoker vs. non-smoker) was systematically recorded, we were unable to reliably quantify cumulative tobacco exposure in terms of pack-years or duration of smoking. This was primarily due to incomplete reporting and substantial variability in patient responses across follow-up visits, with many participants either not providing detailed information or reporting fluctuating smoking behaviors over time. Consequently, dose–response relationships between tobacco exposure and lesion persistence or progression could not be explored. Future studies should incorporate standardized, longitudinal quantification of smoking exposure to better characterize its role in HPV persistence and cervical lesion evolution. Although the study was not preregistered, all predefined outcomes were systematically collected and reported, including both statistically significant and non-significant findings, in order to minimize selective reporting bias. Future studies employing standardized excision techniques and harmonized specimen handling protocols would allow for accurate incorporation of margin involvement into prognostic evaluation.

## 5. Conclusions

This longitudinal study shows that most HPV-related cervical lesions regress within two years, while a clinically important subset, particularly those associated with HPV16/18 and older age, remains at higher risk for persistence and progression. High-grade cytological categories (ASC-H and HSIL) demonstrated strong concordance with histologic outcomes, confirming their diagnostic and prognostic value. The moderate agreement between cytology, colposcopy, and histopathology supports a multimodal, risk-adapted approach integrating HPV genotyping, age, and behavioral factors. Although smoking showed only a non-significant trend toward higher HR-HPV persistence in our cohort, its established biological role supports inclusion of smoking-cessation counseling in cervical cancer prevention strategies.

## Figures and Tables

**Table 1:** Baseline characteristics of the study population (n = 351): Cervical intraepithelial neoplasia, CIN; Atypical squamous cells, cannot exclude HSIL, ASC-H; Atypical squamous cells of undetermined significance, ASC-US; High-grade squamous intraepithelial lesion, HSIL; Low-grade squamous intraepithelial lesion, LSIL. * HSIL (CIN II-III equivocal) refers to cases in which the histopathological report indicated “cannot exclude CIN III; ** HSIL (CIN II-III equivocal) refers to colposcopic impressions in which the severity of the lesion could not be clearly distinguished between CIN II and CIN III at baseline.

Variable	Category			n (%)
Age (years)	Mean ± SD/Median/Mode	43.8 ± 11.4/43/44		351
Age groups	20–29			38 (10.8%)
	30–39			100 (28.5%)
	40–49			106 (30.2%)
	50–59			69 (19.7%)
	60–69			34 (9.7%)
	70–79			4 (1.1%)
Education level	Primary (1–8 classes)			70 (19.9%)
	Secondary (High school)			80 (22.8%)
Residence	Higher education			201 (57.3%)
	Urban			239 (68.1%)
	Rural			112 (31.9%)
Marital status	Single			120 (34.2%)
	Married/Partnered			191 (54.4%)
	Divorced/Widowed			40 (11.4%)
Number of pregnancies	0			85
	1			115
	2			96
	≥3			55
Number of births (parity)	0			105
	1			114
	2			85
	≥3			47
Menopause status	Premenopausal			264 (75.2%)
	Postmenopausal			87 (24.8%)
Hormone use	Yes			0
	No			351
Smoking status	Smokers			249 (70.9%)
	Non-smokers			102 (29.1%)
Baseline cytology	ASC-US			67 (19.1%)
	ASC-H			53 (15.1%)
	LSIL			51 (14.5%)
	HSIL			180 (51.3%)
Baseline HPV status	LR HPV positive			116 (18.01%)
	HR HPV positive			528 (81.99%)
	Total			644
Number of HPV types	No. of HPV genotypes per patient	HR-HPV only	LR-HPV only	Any HPV genotype (HR and/or LR)
	1	184 (52.4%)	93 (26.5%)	146 (41.6%)
	2	104 (29.6%)	10 (2.8%)	137 (39%)
	3	38 (10.8%)	1 (0.3%)	54 (15.4%)
	4	4 (1.1%)	0 (0%)	10 (2.8%)
	5	0 (0%)	0 (0%)	3 (0.9%)
	6	1 (0.3%)	0 (0%)	0 (0%)
	7	0 (0%)	0 (0%)	1 (0.3%)
	Total	528	116	644
Colposcopy With Biopsy	CIN I			3 (0.85%)
	CIN II			128 (36.47%)
	CIN III			121 (34.47%)
	** HSIL (CIN II-III equivocal)			99 (28.2%)
Conization Histopathology	CIN I			28 (7.98%)
	CIN II			97 (27.64%)
	CIN III			140 (39.89%)
	* HSIL (CIN II-III equivocal)			86 (24.5%)

**Table 2:** Integrated Distribution of Cytological Findings and Lesion Outcomes at Baseline, 1-Year, and 2-Year Follow-Up: Atypical squamous cells, cannot exclude HSIL, ASC-H; Atypical squamous cells of undetermined significance, ASC-US; High-grade squamous intraepithelial lesion, HSIL; Low-grade squamous intraepithelial lesion, LSIL.

Baseline Cytological Findings						
Age Group	Negative (0)	ASC-US (1)	ASC-H (2)	LSIL (3)	HSIL (4)	Total
20–29	0 (0%)	5 (1.4%)	6 (1.7%)	10 (2.8%)	17 (4.8%)	38 (10.8%)
30–39	0 (0%)	17 (4.8%)	17 (4.8%)	16 (4.6%)	50 (14.2%)	100 (28.5%)
40–49	0 (0%)	18 (5.1%)	16 (4.6%)	11 (3.1%)	61 (17.4%)	106 (30.2%)
50–59	0 (0%)	18 (5.1%)	8 (2.3%)	10 (2.8%)	33 (9.4%)	69 (19.7%)
60–69	0 (0%)	8 (2.3%)	5 (1.4%)	4 (1.1%)	17 (4.8%)	34 (9.7%)
70–79	0 (0%)	1 (0.3%)	1 (0.3%)	0 (0%)	2 (0.6%)	4 (1.1%)
Total	0 (0%)	67 (19.1%)	53 (15.1%)	51 (145%)	180 (51.3%)	351 (100%)
One-Year Cytological Outcomes						
20–29	29 (8.3%)	4 (1.1%)	5 (1.4%)	0 (0%)	0 (0%)	38 (10.8%)
30–39	63 (17.9%)	22 (6.3%)	11 (3.1%)	2 (0.6%)	2 (0.6%)	100 (28.5%)
40–49	70 (19.9%)	19 (5.4%)	7 (2%)	6 (1.7%)	4 (1.1%)	106 (30.2%)
50–59	39 (11.1%)	20 (5.7%)	6 (1.7%)	3 (0.9%)	1 (0.3%)	69 (19.7%)
60–69	15 (4.3%)	14 (4.0%)	5 (1.4%)	0 (0%)	0 (0%)	34 (9.7%)
70–79	3 (0.9%)	1 (0.3%)	0 (0%)	0 (0%)	0 (0%)	4 (1.1%)
Total	219 (62.4%)	80 (22.8%)	34 (9.7%)	11 (3.1%)	7 (2.0%)	351 (100%)
Two-Year Cytological outcomes						
20–29	23 (6.5%)	13 (3.7%)	2 (0.6%)	0 (0%)	0 (0%)	38 (10.8%)
30–39	71 (20.2%)	27 (7.7%)	2 (0.6%)	0 (0%)	0 (0%)	100 (28.5%)
40–49	82 (23.4%)	23 (6.6%)	1 (0.3%)	0 (0%)	0 (0%)	106 (30.2%)
50–59	53 (15.1%)	13 (3.7%)	3 (0.9%)	0 (0%)	0 (0%)	69 (19.7%)
60–69	19 (5.4%)	12 (3.4%)	3 (0.9%)	0 (0%)	0 (0%)	34 (9.7%)
70–79	4 (1.1%)	0 (0%)	0 (0%)	0 (0%)	0 (0%)	4 (1.1%)
Total	252 (71.8%)	88 (25.1%)	11 (3.1%)	0 (0%)	0 (0%)	351 (100%)

**Table 3:** Cross-tabulation between colposcopic and conization results, showing diagnostic agreement (highlighted in grey), overestimation, and underestimation; Cervical intraepithelial neoplasia, CIN. * HSIL (CIN II-III equivocal) refers to cases in which the histopathological report indicated “cannot exclude CIN III, ** HSIL (CIN II-III equivocal) refers to colposcopic impressions in which the severity of the lesion could not be clearly distinguished between CIN II and CIN III at baseline. The cells highlighted in grey represent the number of cases with matching results between the two diagnostic methods. The two arrows indicate the direction regarding the number of cases that were overdiagnosed or underdiagnosed by conization.

	Conization				
	CIN I	CIN II	CIN III	* HSIL (CIN II-III Equivocal)	Total
Colposcopy	** 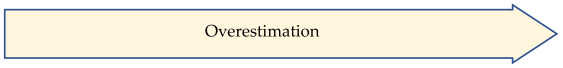 **				
CIN I	0	0	3	0	3
CIN II	16	64	17	31	128
CIN III	7	16	85	12	121
** HSIL (CIN II-III equivocal)	4	17	35	43	99
Total	28	97	140	86	351
	** 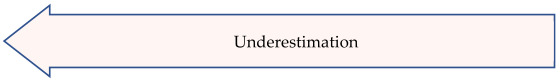 **				

Pearson Chi-Square = 122.4, df = 9, *p* < 0.001; Kappa = 0.345, *p* < 0.001.

**Table 4:** Cross-tabulation between cytologic categories (ASC-US, ASC-H, LSIL, HSIL), colposcopic diagnoses (CIN I, CIN II, CIN III, HSIL) and Pap test (ASC-US, ASC-H, LSIL, HSIL): Cervical intraepithelial neoplasia, CIN; Atypical squamous cells, cannot exclude HSIL, ASC-H; Atypical squamous cells of undetermined significance, ASC-US; High-grade squamous intraepithelial lesion, HSIL; Low-grade squamous intraepithelial lesion, LSIL; Pap, Papanicolaou test. HSIL (CIN II-III equivocal) refers to cases in which the histopathological report indicated “cannot exclude CIN III and to colposcopic impressions in which the severity of the lesion could not be clearly distinguished between CIN II and CIN III at baseline.

	Cytology									
	CIN I		CIN II		CIN III		HSIL (CIN II-III Equivocal)		Total Cases	Main Colposcopic and Main Histologic Correlation (% Within Pap)
Pap Category	Colpo	Histo	Colpo	Histo	Colpo	Histo	Colpo	Histo		
ASC-US	1 (1.5%)	14 (20.9%)	23 (34.3%)	18 (26.9%)	41 (61.2%)	34 (50.7%)	2 (3.0%)	1 (1.5%)	67	Most cases correspond to CIN III (61.2%) → underestimated by Pap
										Predominantly CIN III → underestimation by cytology
ASC-H	0 (0%)	8 (15.1%)	28 (52.8%)	17 (32.1%)	18 (34.0%)	22 (41.5%)	7 (13.2%)	6 (11.3%)	53	Majority CIN II (52.8%) → moderate correlation
										Strong correlation with HSIL (CIN II-III equivocal) → statistically significant (*p* < 0.001)
LSIL	0 (0%)	3 (5.9%)	30 (58.8%)	27 (52.9%)	10 (19.6%)	9 (17.6%)	11 (21.6%)	12 (23.5%)	51	Main match CIN II (58.8%) → tends to underestimate severity
										Predominantly CIN II and HSIL (CIN II-III equivocal) → underestimation trend
HSIL	2 (1.1%)	3 (1.7%)	47 (26.1%)	35 (19.4%)	52 (28.9%)	75 (41.7%)	79 (43.9%)	67 (37.2%)	180	Strong association with HSIL (CIN II-III equivocal) (43.9%) → good diagnostic concordance
										Excellent correlation with CIN III and HSIL (CIN II-III equivocal) → *p* < 0.001

**Table 5:** Association between smoking status and cervical cytological outcomes and HR-HPV persistence during follow-up.

Association Between Smoking Status and Papanicolaou Test Outcomes Over Time			
Outcome	No Persistence/No Progression (n, %)	Persistent/Progressive Disease (n, %)	*p*-Value
Persistence—1 year	Non-smokers: n = 93 (29.4%)	Non-smokers: n = 9 (25.7%)	1 year: 0.646
	Smokers: n = 223 (70.6%)	Smokers: n = 26 (74.3%)	
Persistence—2 years	Non-smokers: n = 97 (31.1%)	Non-smokers: n = 5 (12.8%)	2 years: 0.018
	Smokers: n = 215 (68.9%)	Smokers: n = 34 (87.2%)	
Progression—1 year	Non-smokers: n = 96 (28.0%)	Non-smokers: n = 6 (75.0%)	1 year: 0.004
	Smokers: n = 247 (72.0%)	Smokers: n = 2 (25.0%)	
Progression—2 years	Non-smokers: n = 84 (26.9%)	Non-smokers: n = 18 (46.2%)	2 years: 0.013
	Smokers: n = 228 (73.1%)	Smokers: n = 21 (53.8%)	
Association between smoking status and HR-HPV persistence after baseline excisional treatment			
Persistence—1 year	Non-smokers n = 59 (57.8%)	Non-smokers n = 43 (42.2%)	1 year: 0.272
	Smokers: n = 128 (51.4%)	Smokers: n = 121 (48.6%)	
Persistence—2 years	Non-smokers n = 86 (84.3%)	Non-smokers n = 16 (15.7%)	2 years: 0.131
	Smokers: n = 192 (77.1%)	Smokers: n = 57 (22.9%)	

## Data Availability

The data presented in this study are available on request from the corresponding authors (please specify the reason for restriction, e.g., the data are not publicly available due to privacy or ethical restrictions).

## Abbreviations

AGC	Atypical glandular cells
ASC-H	Atypical squamous cells, cannot exclude HSIL
ASC-US	Atypical squamous cells of undetermined significance
ASIR	Global age-standardized incidence rate
CIN	Cervical intraepithelial neoplasia
DNA	Deoxyribonucleic acid
HPVs	Human papillomaviruses
HR-HPV	High-Risk human papillomavirus
HSIL	High-grade squamous intraepithelial lesion
LEEP	Loop electrosurgical excision procedure
LMICs	Low- and middle-income countries
LR-HPV	Low-Risk human papillomavirus
LSIL	Low-grade squamous intraepithelial lesion
NILM	Negative for intraepithelial lesion or malignancy
OR (95% CI)	Odds ratios with 95% confidence intervals
WHO	World Health Organization
